# Behavior Correlates of Post-Stroke Disability Using Data Mining and Infographics

**DOI:** 10.9734/BJMMR/2016/21601

**Published:** 2015-09-29

**Authors:** Sunmoo Yoon, Jose Gutierrez

**Affiliations:** 1 School of Nursing, Columbia University, New York, USA; 2 Department of Neurology, Columbia University, New York, USA

**Keywords:** Stroke, patient outcome, data mining, visualization

## Abstract

**Purpose:**

Disability is a potential risk for stroke survivors. This study aims to identify disability risk factors associated with stroke and their relative importance and relationships from a national behavioral risk factor dataset.

**Methods:**

Data of post-stroke individuals in the U.S (n=19,603) including 397 variables were extracted from a publically available national dataset and analyzed. Data mining algorithms including C4.5 and linear regression with M5s methods were applied to build association models for post-stroke disability using Weka software. The relative importance and relationship of 70 variables associated with disability were presented in infographics for clinicians to understand easily.

**Results:**

Fifty-five percent of post-stroke patients experience disability. Exercise, employment and satisfaction of life were relatively important factors associated with disability among stroke patients. Modifiable behavior factors strongly associated with disability include exercise (OR: 0.46, P<0.01) and good rest (OR 0.37, P<0.01).

**Conclusions:**

Data mining is promising to discover factors associated with post-stroke disability from a large population dataset. The findings can be potentially valuable for establishing the priorities for clinicians and researchers and for stroke patient education. The methods may generalize to other health conditions.

## 1. INTRODUCTION

In the United States, seven million stroke patients live beyond the acute stroke phase with significant disability and impairment [1]. Most have varying levels of disability [2]. Information provided by clinicians to patients and their families is often focused on etiology or pathophysiological facts such as the size and location of brain lesions [3]. In clinical settings, due to the sudden onset of the disease, stroke patients are often uncertain about their long-term prognosis. Nevertheless, predictors related to long-term stroke outcomes, such as early rehabilitation, smoking, drinking, early stroke recognition, and social support, have been rarely communicated to clinicians [2,4–6]. Moreover, patient-level phenotypes are vital for designing personalized stroke management after an initial incident but are often underused.

To date, methods to investigate post-stroke disability risk factors have been limited to traditional population-level statistics, which allow us to compute only a smaller number of variables or to test a limited number of hypotheses. Stroke research results have been poorly communicated to clinicians who should translate and apply such knowledge at bedside [3,7]. Meanwhile, mining algorithms have been successfully applied to discover medical knowledge from large datasets by investigating hundreds or thousands of variables simultaneously [8]. Data mining has been an established method for studying genomics, phenotypes, pharmacology, or other biomedical problems, [9–17] and have been effectively used to discover correlates of diseases such as hypertension [14], health failure [16], gastrointestinal bleeding [17], diabetes [11], metabolic syndromes [12], and occupational injuries [15]. Infographics have been used to effectively facilitate the intuitive presentation of complex mining studies [18–20].

Data mining studies rarely incorporate clinical domain experts whose decisions are critical for every step of the analysis. Further, data mining studies seldom utilize a conceptual framework to provide guidance for interpreting the and generating hypotheses. In contrast, we incorporated a validated conceptual framework to guide analysis and interpretation, and used clinical expert decision in every analytic step. The purpose of this paper is to present a disability outcomes association model for post-stroke patients based on data mining and an infographics method for presenting such results to clinicians.

## 2. METHODS

### 2.1 Data and Tools

The dataset was obtained in 2011 from the Behavioral Risk Factor Surveillance System (BRFSS), the world’s largest, ongoing health survey released by The Centers for Disease Control and Prevention (CDC) in the United States [21]. The BRFSS comprises de-identified publicly available data, exempt from institutional review board (IRB) approval. We identified total 19,603 patients who were previously diagnosed with stroke (patient self-report, interview in 2010) from 451,075 BRFSS respondents. Data were prepared in SAS and analyzed using Weka v3.7 [22] to build the disability association model.

### 2.2 Conceptual Framework

**Fig. 1:**
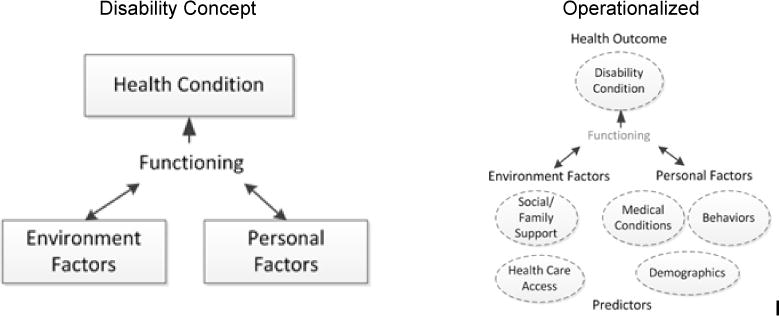
WHO’s international classification of functioning, disability, and health (ICH) (Left), operationalized concepts of ICF (Right)

Our analysis was guided by the World Health Organization (WHO) International Classification of Functioning, Disability and Health (ICH), which defines disability as “decreased function status due to morbidity and injury” (Fig. 1). According to the ICH model, disability is not predefined but dynamic, influenced by personal or environmental factors [23]. BRFSS utilizes this broad concept of disability in stroke-related questionnaires addressing quality of life, health status, and days of experiencing difficulties due to physical or mental health limitations. For example, medical conditions include diabetes and depression, and social factors include social support, family support, and accessibility to emotional support. This framework was applied to organize variables during the analysis phase and to identify the relationships among the factors during the interpretation phase for this study. The outcome variables comprised the functioning related variables in BRFSS the limitation of any usual activities, such as self-care, work or recreation due to physical, mental and emotional problems, such as “during the past 30 days, for how long did poor physical or mental health keep you from doing your usual activities, such as self-care, work, or recreation?”

### 2.3 Data Mining Process

**Fig. 2:**
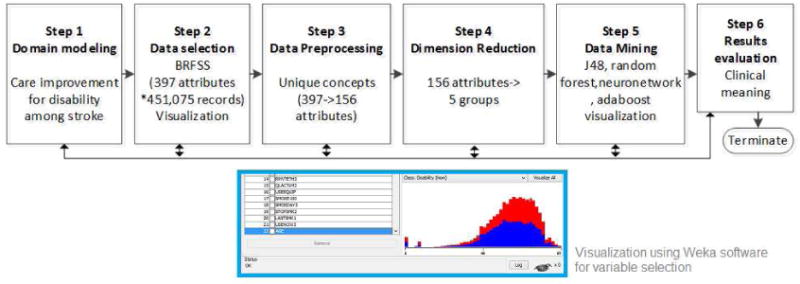
Steps of data mining for building a disability association model for post-stroke patients

As shown in Fig. 2, the iterative process^8^ of analysis consists of the following steps: problem understanding, data understanding, data preparation, model development, model evaluation.

#### 2.3.1 Reducing dimensionality and projecting data

Stroke experts deleted manually duplicate or irrelevant variables (e.g. phone number, disaster preparedness, and dental cleaning), resulting in 156 variables from 397 initial variables. The 397 initial variables were given to each stroke experts independently to identify irrelevant variables, followed by a consensus meeting. Next, stroke experts grouped 156 variables into the following categories based on the conceptual framework: 1) medical conditions, 2) demographic factors, 3) modifiable behavior factors; 4) social support; and 5) access to health care. Next, stroke experts further filtered variables resulting in 139 variables and applied a correlation-based algorithm *CFS attribute evaluator* [22], which evaluates the worth of a subset of variables by considering the individual predictive ability. For the modifiable behavior category, 11 strongly associated variables were further selected by stroke domain experts. For other sub-categories, several iterations of transformation and selection processes resulted in 12 variables for the medical condition category, 11 variables for the demographics category, 4 variables for the social support category, and 4 variables for the health care access category. Missing values (.00%–3.25%) were not replaced by computational imputation.

#### 2.3.2 Association modeling and validation

First, in order to examine overall association of variables, the relative importance of each variable was calculated by linear regression with M5 [24]. M5 method are chosen for this study because it is one of few advanced machine learning schemes to compute the class with continuous variables [25]. M5 splits and prunes recursively performing regression, then greedily drops terms for the cases improving the effort estimates. Not only has M5 method been proven effectively to hand both enumerated attributes and missing values, but also it has advantages of producing compact and comprehensive regression model [24–26]. The calculated relative importance was visualized using Tableau software.

Next, in order to examine detailed information regarding how the variables were related, disability association models of each category were generated. In order to avoid algorithm dependency, several different data mining algorithms suggested as top 10 data mining algorithms were applied first to build the models [27–29]. C4.5 (J48) and Adaboost (AdaboostM1), which are known as being built accurately and based on sound theories, were applied to the data set. The artificial neural network (MultilayerPerceptron) [24,25] were applied to build a association model because it is known as a powerful technique for complex disease and utilized across various scientific disciplines. Although the neural network shows the high accuracy of association, the other algorithms were applied further because the results are technically difficult to understand the hidden layer [26]. We also chose one of the most accurate Random Forest algorithms which runs efficiently on large databases [27]. The model built by C4.5 (J48) was chosen based on model accuracy and the model interpretability. Our selected algorithm, C4.5 (J48) is known as a statistical classifier which builds decision trees using the concept of information entropy. J48 finds the normalized information gain from splitting on each variable, selects the highest, and recursively creates node that splits on the best normalized information gain and add those nods as children [27,28] Unlike Adaboost, artificial neural network and Random forest, C4.5(J48) produces a visualization model in a tree form which is intuitive and relatively easy to understand and transformarable into infographics.

The association models were validated using the cross-validation function in Weka. It automatically divided the dataset into two. The association models were generated from the first subset of data, and tested on the other subset of data. The model’s accuracy (correctly classified instances) was tested by applying a 10-fold cross validation, meaning that our dataset was randomly divided into a training set (90% of cases) and a validation set (10% of cases). We evaluated the model’s performance using proportion correctly classified and the area of under the receiver operating characteristic curve (AUC) [30].

## 3. RESULTS

### 3.1 Characteristics of the Study Population

The characteristics of personal, environmental and the health conditions of the stroke survivors are summarized in Table 1.

The socio-demographic characteristics and the health conditions of the stroke survivors are summarized in Table 1. The mean age was 66.5 (SD=15.2) with 62% being female. The majority of respondents were White (76%) followed by Blacks (11%) and Hispanics (5%) in the U.S. Forty six percent had some college-level education or higher. Fifty one percent were retired, and 15% were employed after their first stroke. In terms of health care access, 7% answered they had no health care coverage. In fact, 14% reported that they could not see doctors due to cost. More than half said their activities were limited due to physical, mental or emotional problems. One third had comorbidities such as myocardial infarction and angina. Approximately 40% were former smokers, while nearly 20% were current smokers. Forty percent were required to use assistive device such as a wheel chair or cane. Twenty eight percent had fallen within 3 months, 44% of whom were injured from the fall.

### 3.2 Association Models

**Fig. 3:**
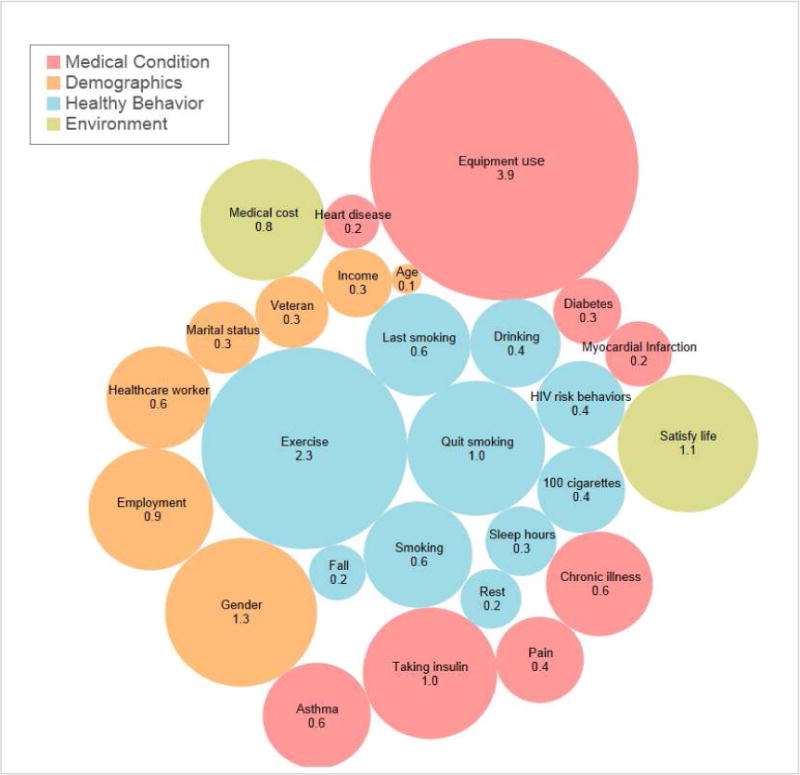
Infographics of correlates of disability among post-stroke patients (number and size representing β calculated by linear regression and M5’s methods representing relative importance using weka software, model fit: correlation coefficeint 0.47, root mean squared error 10.74,)

The overall association of each variable to disability is displayed in Fig. 3. The size of each bubble represents the degree of association to disability calculated by linear regression and M5’s methods using weka software (model fit: correlation coefficient 0.47, root mean squared error 10.74). Exercise appeared to be the strongest association compared to age or disease conditions including heart disease or diabetes.

**Fig. 4:**
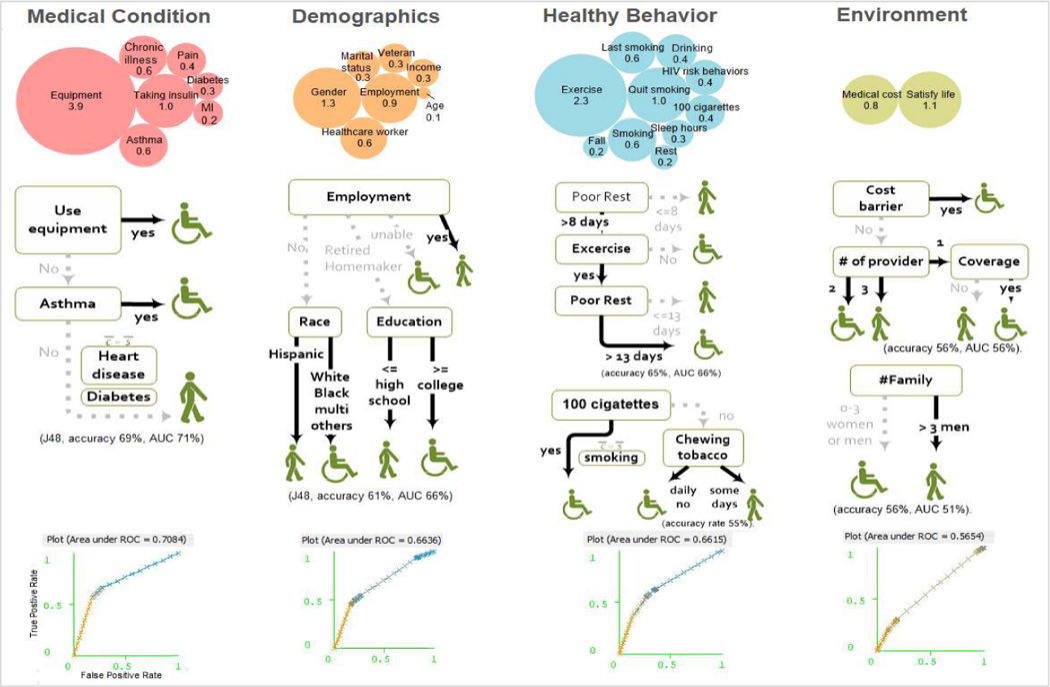
Infographics of correlation models for disability among post-stroke patients generated using C4.5 (J48) algorithm using Weka software

The infographic in Fig. 4 illustrates different categories of associations generated C4.5 algorithm: 1) medical conditions, 2) demographics, 3) modifiable behaviors, 4) health care access, and 5) social and family support. The first three are personal factors and the latter two are environmental factors. Table 2 summarizes the results.

#### 3.2.1 Personal factor-medical conditions

The medical condition category included variables related to heart attack, angina, cancer, snoring, depression, asthma, diabetes, and insulin use. Use of assistive device (e.g., a cane or a walker) and asthma appeared as the correlates of disability among stroke survivors (model accuracy 69%, AUC 71%). As previously mentioned, the outcome variable (disability) in this study depicted the one whose usual activities such as self-care, work or recreation are affected due to mental, emotional and physical problems over 15 days per month. Those diagnosed with asthma were 1.5 times more likely to have disabilities (probability 0.57 vs 0.37, OR: 2.13, 95% CI: 1.93 to 2.35, P<0.0001).

#### 3.2.2 Personal factor-demographics

The analysis revealed employment status as the strongest correlates among demographic factors, compared to other socio-economic determinants such as income, education and ethnicity/race (model accuracy 61%, AUC 66%). Half of retired stroke patients (51%) are 1.17 times more likely to have disability if their income level is less than $25,000 per year (probability 0.55 vs 0.47, OR 1.29, 95% CI: 1.26 to 1.51, P <0.0001). Stroke survivors with higher education were 1.10 times more likely to have disability among the retired stroke survivors (probability 0.53 vs 0.48, OR 1.21, 95% CI: 1.12 to 1.31, P<0.0001).

#### 3.2.3 Personal factor-modifiable behaviors

Quality of rest and exercise appeared as the stronger indicators of disability among stroke patients from the 87 behavior risk variables (model accuracy 65%, AUC 66%). Stroke survivors who had good rest (poor rest <=8 days per month, meaning enough rest) were less likely to have disability (probability of disability 0.48 vs 0.71, OR 0.37, 95% CI 0.35 to 0.40, P<0.0001). This 8-day threshold of having a poor quality rest goes up to 13 days for stroke patients who regularly exercise. Stroke patients who regularly exercise were less likely to have disability (probability of disability 0.47 vs 0.66, OR 0.46, 95% CI 0.43–0.49, P<0.0001) We also magnified smoking status because smoking has been one of the most studied topic in this domain and is currently most emphasized in clinical practice with regulation and policies although it was not a strong predictor of stroke outcome (model accuracy 55%). BRFSS included smoking related variables such as total number of cigarettes, frequency of smoking, willingness to stop smoking, last time smoked, frequency of using chewing tobacco, snuff, or snus. Whether the total number of cigarettes smoked in an entire life was less than 100 cigarettes (5 packs) was a stronger indicator than frequency or period of smoking cessation. Stroke survivors who smoked 100 cigarettes in their entire life were more likely to have disabilities regardless of being a former smoker or a daily smoker.

#### 3.2.4 Environmental factor-health care access

Stroke survivors who could not see a doctor when needed due to cost during the past 12 months, were more likely to have disabilities. Among those without cost barriers who had a primary health provider, stroke survivors with no health insurance were less likely to have disabilities (model accuracy 56%, AUC 56%).

#### 3.2.5 Environmental factor-social or family support

Compared to the fact that the number of adult women in a family did not influence the outcome, the number of men in a family (> 3 men in a family) was associated with the positive stroke outcome (model accuracy 56%, AUC 51%). The infographics showed that disability was increased when frequency of social support is decreased.

## 4. DISCUSSION

A data mining approach was used to discover the degree of association over hundreds of risk factors related to disability for stroke population from a national dataset. Our novel mining approach executed by clinical domain experts and using a conceptual framework to organize the data mining process adds new knowledge of the relative importance and relationship of 70 variables associated with disability to the field. This can help establishing the priorities to focus on for clinicians and stroke researchers. This study introduced relatively unknown factors of stroke disability such as employment, quality of rest, and asthma status as a new knowledge. In addition, this study complements the known risk factors of stroke disability (e.g., exercise, sleep, diabetes, smoking, heart disease and age) with the models explaining the relationship of the variables. Moreover, this study provides additional information of contradictory correlates such as race and ethnicity. This will be further discussed below.

Data mining process executed by stroke domain experts efficiently generated clinically suitable association models for disability from hundreds of variables, which possibly contain thousands of theoretical combinations of conditions. Modern data mining in its nature requires clinical domain expertise in each step, from in-depth problem understanding to results interpretation, in order to find clinically meaningful and applicable new knowledge. For this quest, this study offers insights for clinicians about how to apply emerging modern techniques using free-software and publically available data for other health conditions. Next we discuss three interesting findings of risk factors of disability.

First, in demographics, employment status was identified as a primary factor associated with disability. Despite to the benefits such as empowerment, sense of self-control and happiness, only half of stroke patients are usually able to go back to work [31,32]. In this study, stroke patients answering employed (15%) were more likely not to have disability. Our finding may consider for multidisciplinary stroke care teams paying attention for the patients’ employment status, [33] considering the fact that the more younger people are attacked by stroke [31]. Further, Hispanics were less likely to have disabilities than others among unemployed stroke survivors. Mixed results have been reported regarding racial disparities and stroke outcomes [34–36]. Our study provides evidence that Hispanics have a better outcome than others among the unemployed stroke population.

Second, in medical conditions, asthma showed as strong correlates of disability. Asthma status was the strong predictor regardless of status of heart disease or diabetes. Even if individuals with heart diseases such as coronary artery disease or myocardial infarction, stroke patients without asthma were less likely to have disability. Although association between asthma and cardiovascular diseases has been reported in several studies, the association between asthma and stroke has been rarely reported [37]. In terms of diabetes and stroke, contradictory results have been shown in previous studies [38]. Our study finding further explains that asthma was a stronger predictor than diabetes. The association with asthma and stroke were relatively unknown; this may be a new avenue to explore.

Third, in terms of modifiable lifestyles, strong evidence has been supported positive outcomes of self-management programs after stroke [31,39]. A recent multicenter randomized controlled study also emphasized the importance of such programs to improve stroke outcome. Our study finding regarding quality of rest and exercise as the main correlates among over a hundred behaviors is one of our unique contributions to stroke self-management programs. In particular ‘8-day threshold increased to 13 days for the one who regularly exercises’ may feed the body of knowledge of such programs. In terms of smoking, stroke survivors who smoked 100 cigarettes in their entire life were more likely to have a disability regardless of being a former smoker or an every day smoker. This contains clinical implication that accurate assessment of the amount of smoking in the practice may be needed.

Our study has several limitations. We used cross-sectional data, so the results should cautiously be interpreted and do not represent causality. Despite its comprehensiveness, BRFSS lacks diet-related variables, which may be related to outcome. In addition, all stroke respondents were limited to those who were at least able to answer a telephone survey and willing to complete the survey, which excluded patients with greater stroke severity. Further, subtypes of stroke were not taken into account in this study because of unavailability of the variable. In addition, only a few common data mining association methods such as C4.5, Adaboost, Neuroal network and RandomForest) were applied. Further studies applying different machine learning algorithms (e.g. ensemble methods) with longitudinal dataset will strengthen the results.

## 5. CONCLUSION

Association data mining may not only offer implications for clinicians but also help generate new hypotheses regarding stroke outcomes. Simple infographics may enhance and comprehensibility of the study results for clinicians, and have potential for patient education.

## Figures and Tables

**Table 1:** Characteristics of stroke survivors in 2011 a national survey (N=19,458)

Characteristics	Sample	Characteristics	Sample

Personal factors		Personal factors	
**Demographics**		**Modifiable behaviors**	
Age, years, mean	66.5 (SD=15.2)	**Smoking**	
Sex (female)	12,137 (62%)	Current everyday	2,704 (14%)
**Race/ethnicity**		Current someday	1,041 (5%)
White	14,825 (76%)	Former smoke	7,480 (39%)
Black	2,103 (11%)	Never smoke	8,100 (42%)
Other	675 (3%)	**Fall in 3 months**	
Multiracial	532 (3%)	Yes	5,455 (28%)
Hispanic	985 (5%)	No	12,484 (64%)
**Education level**		**General Health**	
Never attend	42 (0%)	Excellent	756 (4%)
Grades 1–8 years	1,246 (6%)	Very good	2,744 (14%)
Grades 9–11 years	2,226 (12%)	Good	5,499 (28%)
Grade 12	6,923 (36%)	Fair	5,699 (29%)
College 1–3 years	5,071 (26%)	Poor	4,637(24%)
College >= 4 years	3,919 (20%)	**Health not good past month**	
**Employment**		Physical, days, mean	11 (SD=12.5)
Employed for wages	2,208 (11%)	Mental, days, mean	6 (SD=10.2)
Self-employed	705 (4%)	Both, days, mean	10 (SD=11.2)
Out of work > 1 year	543 (3%)	**Disability**	
Out of work < 1 year	306 (2%)	Yes	10,827 (55%)
A homemaker	1,238 (6%)	No	8,631 (44%)
A student	58 (0%)	Not sure	125 (1%)
Retired	9,909 (51%)	Refused	19 (0%)
Unable to work	4,382 (23%)		
**Marital Status**		**Equipment use**	
Married	8,049 (41%)	Yes	7,537 (39%)
Divorced	3,580 (18%)	No	11,894 (61%)
Widowed	5,790 (30%)		
Separated	536 (3%)	**Environmental factors**	
Never Married	1,249 (7%)	*Health care access*	
**Medical conditions**		Have coverage	18,085 (93%)
Myocardial infarction	5,731 (30%)	No coverage	1,318 (7%)
Coronary heart disease	4,890 (26%)	**Not see doctor due to cost**	
Asthma	3,826 (20%)	Cost barrier	2,775 (14%)
Injury by fall	2,371 (44%)	No cost barrier	16,605 (86%)

**Table 2:** Variables associated with disability among stroke survivors

Category	Variable	Rank*	Model†		Literature

Personal factors					
**Medical conditions**	Use of assistive device	1	☒	accuracy	
	Insulin use	6		69%	
	Asthma	10	☒	AUC 73%	
	Chronic illness	11			
	Pain	16			[40]
	Daibetes	22	☒		[41–44]
	Myocardial infarction	23	☒		[41–43]
	Coronary heart disease	26	☒		
	Cancer				
	Snoring				[45]
	Depression				[40,46]
**Demographics**	Gender	3	☒	accuracy	[41–44,47,48]
	Employment	7	☒	61%	[41,46,48]
	Marital status	18		AUC 66%	[48]
	Veteran experience	19			
	Income level	21	☒		[43]
	Age	27			[41–44,47,48]
	Race	28	☒		[41]
	Education		☒		[47,48]
**Modifiable behaviors**	Excercise	2	☒	accuracy	[47]
	Smoking	5	☒	65%	[5,41,43,47]
	Quit smoking	5		AUC 66%	[46]
	Last smoking	12			
	Drinking	14			[41–43]
	HIV risk behavior	15			
	100 cigarettes in life	17	☒		
	Sleep duration	20			[40,46,49]
	Quality of resting	24	☒		[49]
	# of fall in 3 months	25			
	Preventative screening				
	Immunization behaviors				
**Environmental factors**					
Health care access	Medical cost barriers	8	☒	accuracy	
	Insurance coverage		☒	56%	[50]
	# of health care providers		☒	AUC 56%	
Social/family support	Satisfy of life	4		accuracy 56% AUC 51%	[45]
	Frequency of emotional support				[40]
	# of adults in a family		☒		[44]

* Rank of relative importance calculated by data mining linear regression with M5’s methods range from 1 to 28. Blank means the variable were not seleced by M5 algorithm.

† Variables included in the association model for stroke disability detected by C4.5 algorithm. The association models are presented in Fig. 3
